# Spatial Tissue Proteomics Quantifies Inter- and Intratumor Heterogeneity in Hepatocellular Carcinoma (HCC)*[Fn FN2]

**DOI:** 10.1074/mcp.RA117.000189

**Published:** 2018-01-23

**Authors:** Katarzyna Buczak, Alessandro Ori, Joanna M. Kirkpatrick, Kerstin Holzer, Daniel Dauch, Stephanie Roessler, Volker Endris, Felix Lasitschka, Luca Parca, Alexander Schmidt, Lars Zender, Peter Schirmacher, Jeroen Krijgsveld, Stephan Singer, Martin Beck

**Affiliations:** From the ‡European Molecular Biology Laboratory, Structural and Computational Biology Unit, Heidelberg, Germany;; §Leibniz Institute on Aging – Fritz Lipmann Institute (FLI), Jena, Germany;; ¶European Molecular Biology Laboratory, Proteomics Core Facility, Heidelberg, Germany;; ‖Institute of Pathology, University Hospital Heidelberg, Heidelberg, Germany;; **Department of Internal Medicine VIII, University Hospital Tübingen, 72076 Tübingen, Germany;; ‡‡Department of Physiology I, Institute of Physiology, Eberhard Karls University Tübingen, 72076 Tübingen, Germany;; §§Translational Gastrointestinal Oncology Group, German Consortium for Translational Cancer Research (DKTK), German Cancer Research Center (DKFZ), Heidelberg 69120, Germany;; ¶¶Biozentrum, University of Basel, Basel, Switzerland;; ‖‖European Molecular Biology Laboratory, Genome Biology Unit, Heidelberg, Germany;; *^a^*German Cancer Research Center (DKFZ), Heidelberg, Germany;; *^b^*European Molecular Biology Laboratory, Cell Biology and Biophysics Unit, Heidelberg, Germany

## Abstract

The interpatient variability of tumor proteomes has been investigated on a large scale but many tumors display also intratumoral heterogeneity regarding morphological and genetic features. It remains largely unknown to what extent the local proteome of tumors intrinsically differs. Here, we used hepatocellular carcinoma as a model system to quantify both inter- and intratumor heterogeneity across human patient specimens with spatial resolution. We defined proteomic features that distinguish neoplastic from the directly adjacent nonneoplastic tissue, such as decreased abundance of NADH dehydrogenase complex I. We then demonstrated the existence of intratumoral variations in protein abundance that re-occur across different patient samples, and affect clinically relevant proteins, even in the absence of obvious morphological differences or genetic alterations. Our work demonstrates the suitability and the benefits of using mass spectrometry-based proteomics to analyze diagnostic tumor specimens with spatial resolution. Data are available via ProteomeXchange with identifier PXD007052.

Inter- and intratumoral heterogeneity is a major challenge in personalized medicine, because it directly affects the robustness of diagnostic, prognostic, and therapeutic biomarker predictions (1). Even within a defined tumor entity, the variation of biomarker expression between different patients and across different tumor regions of the same individual specimen (*e.g.* center *versus* periphery) needs to be considered. Particularly the latter is of immediate clinical importance, when only a small tumor fraction can be obtained in the setting of a diagnostic/pretreatment biopsy, and thus the region of withdrawal could directly impact the acquired expression profile. Routine diagnostics of tumors involves evaluation of histomorphological features by conventional microscopy. Although it is often combined with immunohistochemical staining (IHC)^1^ of marker proteins, the number of proteins that can be quantitatively analyzed by IHC is rather limited by the availability of suitable antibodies and the experimental throughput. Mass spectrometry-based proteomics enables the quantitative analysis of protein abundances on a proteome-wide scale, but the majority of previous proteomic analyses of cancer specimens have only focused on the bulk tumor, not taking the spatial context within an individual specimen into account (2, 3).

Formalin-fixed and paraffin-embedded (FFPE) cancer tissue offers the best possible material to routinely study intratumor heterogeneity (ITH) because: (1) FFPE specimens provide excellent integrity of the tissue architecture (superior to frozen specimens), which allows, in combination with Laser Capture Microdissection (LCM), the precise and reproducible spatial separation of local tissue regions; (2) human FFPE specimens are the central part of the clinico-pathological workflow and reflect the standard processing of tissue specimens in pathological routine diagnostics worldwide; (3) FFPE tissues are intrinsically linked to clinical records and often associated with additional pathological data (genomics, *in situ* hybridization, immunohistochemistry etc.).

Mass spectrometry-based proteomics has been already used to study FFPE cancer tissue specimens (4, 5), but rarely in combination with spatial resolution (6–8), because the amount of material that can be obtained from a specific region of an FFPE specimen limits the comprehensiveness, *i.e.* number of identified and quantified proteins. Therefore, the great advantage of excellent spatial preservation of FFPE material has not yet been exploited to systematically and jointly analyze inter- and intratumoral heterogeneity across multiple specimens.

Here, we describe a universal workflow that is based on LCM to separate different tumor regions, followed by ultrasensitive and rapid peptide isolation using the paramagnetic bead technology named SP3 (9), and high-resolution quantitative mass spectrometry (qMS). This workflow enables the reproducible proteomic analysis of FFPE material with very good proteomic coverage and spatial resolution. To demonstrate the power of this workflow, we investigated both inter- and intratumoral proteomic heterogeneity in hepatocellular carcinoma (HCC). HCC is the 5th most frequent cancer worldwide (10), represents the 2nd most frequent cause of cancer related death, and shows a rapidly rising incidence rate, with an annual increase of 2% in the US (11). The therapeutic options for HCC patients are still limited with less than 20% of HCC patients being amenable for a curative treatment (partial hepatectomy or liver transplantation). Accordingly, the prognosis of symptomatic HCC patients is extremely poor, with a five-year-survival of less than 5%. Important insights into the molecular basis and diversity of HCC have been provided by genomic and transcriptomic approaches (12–14), including prediction of tumor relapse (15). Nevertheless, disease relevant alterations at the proteomic level (directly shaping the tumor phenotype), particularly in a spatial context, remain poorly defined. Notably, the heterogeneity of HCCs is less apparent at the histomorphological level, in contrast to other tumor entities, such as nonsmall cell lung cancer (NSCLC).

Here we analyzed the spatial abundance of thousands of proteins across different tumor sectors, nontumorous tissue and across interindividual HCC samples. We show that spatial proteomic analysis accurately recovers known factors associated with HCC, but also identifies novel potential therapeutic candidates. We demonstrate that various, often functionally related proteins, display heterogeneous expression within a tumor that is not reflected on the genetic or gene expression level. Among these, there are known prognostic markers for HCC. Thus, dissecting the “submorphological” inter- and intratumor spatial proteomic heterogeneity may critically support and improve conventional diagnostic procedures, prognosis estimation and ultimately therapeutic options in precision liver oncology.

## MATERIALS AND METHODS

### 

#### 

##### Experimental Design and Statistical Rationale

To investigate the HCC proteome, 5 tumors from different patients were analyzed by two TMT-10plex experiments. First we compared tumor *versus* adjacent nontumoral tissue and then we analyzed difference between tumor center and periphery. For differential protein expression, each patient-sample was treated individually. Protein ratios were calculated for all the protein groups quantified with at least 2 proteotypic peptides. A two components model was fitted on centered ratio distributions. Protein groups with a q value < 0.2 were considered as differential expressed between the conditions tested.

For murine HCC proteome we used label-free quantification (LFQ) approach to analyze extracted tumors from 11 mouse models (1 sample per condition) of different genetic background. Those were compared with the average LFQ values measured from normal liver samples obtained from three different mice.

##### Source of Tissue Specimens

Formalin-fixed and paraffin-embedded tumor tissues were provided by the tissue bank of the National Center for Tumor Diseases (NCT, Heidelberg, Germany) in accordance with the regulations of the tissue bank and the approval of the Ethics Committees of Heidelberg University and the European Molecular Biology Laboratory. Only tissue specimens of high quality (high tumor cell content, lack of significant necrotic and fibrotic changes) as judged by trained pathologist were included and each selected tumor was re-evaluated regarding its grading. Patient characteristics (age, sex, pT-stage, tumor grading, and etiology) are provided in supplemental Table S1. Fresh frozen tissue samples of murine HCCs were generated by D.D. and L.Z. Mouse models were generated by transposon-based gene transfer of different oncogenes (*N-ras^G12V^*, *Myc*, and myristolated *Akt1*) into wildtype mice as well as into mice with homozygous or heterozygous deletions of the tumor suppressor genes *CDKN2a^ARF^* and/or *Trp53* (16).

##### Laser Microdissection of Human HCC Specimens

The specimens were cut on a microtome into 10 μm thick sections and processed as follows: sections were mounted on membrane slides (PEN-membrane, 1 mm glass, Carl Zeiss MicroImaging GmbH, Bernried, Germany), deparaffinized in 2× xylene for 3 min, rehydrated in 2 × 100% ethanol for 2 min, and then washed in 90% (v/v), 70% (v/v) and 50% (v/v) ethanol, stained for 15 s in cresyl violet acetate (1% (w/v) in ACS-grade ethanol (Sigma-Aldrich, Munich, Germany)). Subsequently, the slides were washed in 50% (v/v), 70% (v/v), 90% (v/v) and 100% ethanol and incubated for 5 min in xylene. After air-drying the slides were mounted on the stage of an inverded microscope being a component of a Microbeam LMPC System (Carl Zeiss MicroImaging GmbH). We employed the RoboLPC method to microdissect and capture the different tumor sectors, capsule and nontumorous tissue. For each sector we collected ∼40 mm^2^ of tissue (400 nL).

##### Immunohistochemistry (IHC) Staining and Evaluation

Immunohistochemical stainings were performed with an automated immunostaining instrument (BenchMark ULTRA IHC/ISH Staining module, Ventana Medical Systems, Tucson, AR). The OptiView DAB IHC Detection Kit (OptiView, Ventana Medical Systems) was used based on the manufacturer's protocol. The procedure included the following steps: 4 min deparaffinization at 62 °C, rinsing with EZ Prep (Ventana Medical Systems), incubation with Cell Conditioner No. 1 (Ventana Medical Systems) for 40 min at 90 °C. Primary antibody treatment with following antibodies: RAC1 (GeneTex, Irvine, CA) diluted 1:25, Decorin (Thermo Scientific, Offenbach, Germany) diluted 1:300, HEPAR 1 (Cell marque, Rocklin CA, USA) and Ki67 (clone MIB1, DAKO, Glostrup, Denmark) diluted 1:200 - 24 min treatment at 36 °C, 4 min exposure to Optiview Peroxidase Inhibitor, 12 min incubation with Optiview HQ Universal Linker, 12 min treatment in Optiview HRP Multimer, 8 min incubation with a mixture of Optiview H2O2 and DAB, 4 min exposure to Optiview copper, counterstaining with Hematoxylin for 12 min, 4 min incubation with Bluing Reagent. The incubations were followed by multiple rinsing steps in reaction buffer (Ventana Medical Systems). Dehydration of each FFPE slide was performed as follows: 1 × 5 min 70% (v/v) ethanol, 1 × 5 min 96% (v/v) ethanol, 2 × 5 min 100% ethanol, 1 × 5 min Xylene by using the Leica autostainer XL. Finally the slides were mounted with cover slips (Leica CV5030).

##### Quantitative Proteomics of HCC Specimens

##### Protein Solubilization for FFPE Samples

Tissue sections were collected in PCR tubes containing 100 μl of protein solubilization buffer (80 μm Tris pH 8.0, 80 μm DTT and 4% (w/v) SDS) and processed directly. Samples were sonicated using a Bioruptor Plus (Diagenode) for 25.2 min (15 cycles: 1 min on, 30 s off) at the highest settings, and then boiled for 1 h at 99 °C. Sonication followed by boiling was performed twice. Cysteine residues were alkylated by adding 200 mm iodoacetamide to a final concentration of 15 mm (incubated for 30 min at room temperature in the dark). Reaction was quenched by addition of 10 μl of 200 mm DTT.

##### Protein Purification, Digestion, and Peptide Desalting for FFPE Samples

Sera-Mag Speed Beads (#45152105050250 and #65152105050250, Thermo Scientific) were mixed 1:1, rinsed with water and stored as a 40 μg/μl stock solution in 4 °C, as described in (9). Four μl of beads stock was added to the reaction tube and mixed by pipetting then 100% acetonitrile was added to a final concentration of 50% (v/v). Samples were incubated for 8 min at room temperature to allow protein bindings to the beads. Next, tubes were placed on the magnetic rack. Supernatant was removed and discarded. Beads were washed twice with 180 μl of 70% (v/v) ethanol and once with 180 μl of 100% acetonitrile. After removal of acetonitrile beads were air-dried for 60 s and then resuspended in 7 μl of digestion buffer: 6 μl 4 m urea in 100 mm ammonium bicarbonate (or 100 mm HEPES pH 8.5 in TMT experiment) and 1 μl of 0.1 μg/μl of LysC (Wako). Samples were sonicated for 5 min in water bath, incubated for 5 min at 37 °C and then mixed by pipetting. Digestion was allowed to proceed for 4 h at 37 °C. After the first step of digestion, beads were resuspended by pipetting, urea was diluted to the final concentration of 1.5 m and 1 μl of 1 μg/μl of sequencing grade trypsin (Promega) (1 μg/μl of LysC for TMT-6plex experiment) was added to samples. Digestion was performed for 12 h at 37 °C. After digestion, beads were resuspended by pipetting. 100% acetonitrile was added to the final concentration of 95% (v/v) and samples were incubated for 8 min at room temperature. Tubes were placed on the magnetic rack and washed twice with 100% acetonitrile. Supernatant was removed and beads air-dried and reconstituted in 9 μl of 2% DMSO followed by 5 min of sonication in the water bath. Samples were resuspended by pipetting and placed on the magnetic rack. Supernatant containing peptides was transferred to a fresh tube and acidified with 1 μl of 1% (v/v) formic acid.

##### TMT Labeling

TMT-10plex (5-tumor analysis, Thermo Scientific) or TMT-6plex (additional specimen) reagents were reconstituted in 100% ACN according to the manufacturers instructions. 1 μl of 1 m HEPES pH 8.5 was added to 9 μl of digested and purified peptides. TMT labeling was performed by addition of 1 μl of the TMT reagent. After 30 min of incubation at room temperature, a second portion of TMT reagent (1 μl) was added and incubated for another 30 min. Reaction was quenched with 1 μl of 20 mm lysine in 100 mm ammonium bicarbonate. 4 μl of beads stock solution was added to the sample. Peptides were bound to the beads, washed and eluted as described in peptide purification section. Labeled peptides were pooled together and fractionated.

##### High pH Peptide Fractionation for TMT Labeled Samples

Offline high pH reverse phase fractionation was performed using an Agilent 1200 Infinity HPLC System equipped with a quaternary pump, degasser, variable wavelength UV detector (set to 254 nm), peltier-cooled autosampler, and fraction collector (both set at 10 °C for all samples). The column was a Gemini C18 column (3 μm, 110 Å, 100 × 1.0 mm, Phenomenex) with a Gemini C18, 4 × 2.0 mm SecurityGuard (Phenomenex) cartridge as a guard column. The solvent system consisted of 20 mm ammonium formate (pH 10.0) as mobile phase (A) and 100% acetonitrile as mobile phase (B). The separation was accomplished at a mobile phase flow rate of 0.1 ml/min using the following linear gradient for TMT-6plex experiment: 99% A for 2 min, from 99% A to 37.5% B in 61 min, to 85% B in a further 1 min, and held at 85% B for an additional 5 min, before returning to 99% A and re-equlibration for 18 min. Thirty seven fractions were collected along with the LC separation that were subsequently pooled into 16 fractions. For TMT-10plex experiment, a slightly modified gradient was used, whereby the LC separation time was 100 min from 10% to 40% B and 48 fractions were collected over this separation time, which were again subsequently pooled into 16 fractions. Pooled fractions were dried in a SpeedVac and then stored at −80 °C until LC-MS/MS analysis.

##### Data Acquisition and Processing for TMT Labeled Samples

For TMT-6plex experiments, fractions were resuspended in 10 μl reconstitution buffer (5% (v/v) acetonitrile, 0.1% (v/v) TFA in water) and 7 μl were injected. Peptides were separated using the nanoAcquity UPLC system (Waters) fitted with a trapping (nanoAcquity Symmetry C18, 5 μm, 180 μm × 20 mm) and an analytical column (nanoAcquity BEH C18, 2.5 μm, 75 μm × 500 mm). The outlet of the analytical column was coupled directly to an Orbitrap Fusion (Thermo Fisher Scientific) using the Proxeon nanospray source. Solvent A was water, 0.1% (v/v) formic acid and solvent B was acetonitrile, 0.1% (v/v) formic acid. The samples were loaded with a constant flow of solvent A at 5 μl/min, onto the trapping column. Trapping time was 6 min. Peptides were eluted via the analytical column at a constant flow of 0.3 μl/min, at 55 °C. During the elution step, the percentage of solvent B increased in a linear fashion from 5% to 7% in 10 min, then from 7% B to 30% B in a further 105 min and to 45% B by 130 min. The peptides were introduced into the mass spectrometer via a Pico-Tip Emitter 360 μm OD x 20 μm ID; 10 μm tip (New Objective) and a spray voltage of 2.2kV was applied. The capillary temperature was set at 300 °C. Full scan MS spectra with mass range 300–1500 *m/z* were acquired in profile mode in the Orbitrap with resolution of 60,000 FWHM (at 200 m/z) using the quad isolation. The RF on the S-lens was set to 60%. The filling time was set at maximum of 50 ms with an AGC target of 4 × 10^5^ ions and 1 microscan. The peptide monoisotopic precursor selection was enabled along with relaxed restrictions if too few precursors were found. The most intense ions (instrument operated in Top Speed mode) from the full scan MS were selected for MS2, using quadrupole isolation and a window of 1.6 Da. CID was performed in the ion trap with normalized collision energy of 35%, with an intensity threshold of 5 × 10^3^. A maximum fill time of 70 ms for each precursor ion was set, with an AGC target of 1 × 10^4^ ions and 1 microscan. MS2 data were acquired in centroid with the rapid scan mode. Only multiply charged (2+ to 7+) precursor ions were selected for MS2. The dynamic exclusion list was with a maximum retention period of 40 s and relative mass window of 7 ppm, with isotopes were excluded. The instrument was allowed to inject ions for all available parallelizable time. For the MS3, the precursor selection window was set to the range 400–1300 *m/z*, with an exclude width of 30 *m/z* (high) and 5 *m/z* (low). Isobaric tag loss exclusion was set to Reagent = TMT. The most intense fragments from the MS2 experiment were co-isolated (isolation window 2Da, using Synchronus Precursor Selection = 10) and fragmented by HCD (collision energy, 65%). MS3 spectra were acquired in the Orbitrap over the mass range 100–200 *m/z* and resolution set to 30000. The maximum injection time was set to 100 ms with an AGC target of 1 × 10^5^ ions and 1 microscan. Data were acquired in profile mode and the instrument was allowed to inject ions for all available parallelizable time.

A similar strategy was used for the acquisition of TMT-10plex experiment, with the following exceptions: The analytical column used for the LC separation was 250 mm and the MS data acquisition took place on an Orbitrap Fusion Lumos (Thermo Fisher). Full scan MS spectra with mass range 375–1500 *m/z* were acquired in profile mode in the Orbitrap with resolution of 60,000 FWHM (at 200 m/z) using the quad isolation. The RF on the S-lens was set to 40%. The filling time was set at maximum of 100 ms. The most intense ions (instrument operated for a 3 s cycle time) from the full scan MS were selected for MS2, using quadrupole isolation and a window of 1 Da. HCD was performed with collision energy of 35%. A maximum fill time of 50 ms for each precursor ion was set. MS2 data were acquired with fixed first mass of 120 *m/z*. The dynamic exclusion list was with a maximum retention period of 60 s and relative mass window of 10 ppm. The instrument was not set to inject ions for all available parallelizable time. For the MS3, the precursor selection window was set to the range 400–2000 *m*/*z*, with an exclude width of 18 *m/z* (high) and 5 *m/z* (low). The most intense fragments from the MS2 experiment were co-isolated (using Synchronus Precursor Selection = 8). MS3 spectra were acquired in the Orbitrap over the mass range 100–1000 *m/z* and resolution set to 50,000 FWHM (at 200 m/z). The maximum injection time was set to 105 ms and the instrument was set not to inject ions for all available parallelizable time.

TMT-6plex data were processed using Proteome Discoverer v1.4 (Thermo Fisher Scientific). Data were searched against Uniprot Human fasta database (release 2014_07, 20230 entries) using Mascot v2.2.7 (Matrix Science) with the following settings: Enzyme was set to LysC, with up to 1 missed cleavage. MS1 mass tolerance was set to 10 ppm and MS2 to 0.5 Da. Carbamidomethyl cysteine was set as a fixed modification and oxidation of Methionine as variable. Other modifications included the TMT-6plex modification from the quan method used. The quan method was set for reporter ions quantification with HCD and MS3 (mass tolerance, 20 ppm). The false discovery rate for peptide-spectrum matches (PSMs) was set to 0.01 using Percolator (17).

TMT-10plex data were processed using Proteome Discoverer v2.0 (Thermo Fisher Scientific). Data were searched against Swissprot Human fasta database (release 2016_11, 20211 entries) using Mascot v2.5.1 (Matrix Science) with the following settings: Enzyme was set to trypsin, with up to 1 missed cleavage. Other settings were as for TMT-6plex search data, with the exception of the modifications from the quan method, which was set to TMT10 and Acetyl (Protein N-term) as a variable modification.

Reporter ion intensity values for the filtered PSMs were exported and processed using in-house written R scripts to remove common contaminants and decoy hits. Additionally only PSMs having reporter ion intensities above 1 × 10^3^ in all the relevant TMT channels were retained for quantitative analysis.

##### Data Acquisition and Processing for DIA Samples

Chromatographic separation of peptides was carried out using an EASY nano-LC 1000 system (Thermo Fisher Scientific), equipped with a heated RP-HPLC column (75 μm × 50 cm) packed in-house with 1.9 μm C18 resin (Reprosil-AQ Pur, Dr. Maisch). Peptides were analyzed per LC-MS/MS run using a linear gradient ranging from 95% solvent A (0.15% formic acid, 2% acetonitrile) and 5% solvent B (98% acetonitrile, 2% water, 0.15% formic acid) to 30% solvent B over 120 min at a flow rate of 200 nL/min. Mass spectrometry analysis was performed on a Q-Exactive HF mass spectrometer equipped with a nanoelectrospray ion source (both Thermo Fisher Scientific) and a custom made column heater set to 60 °C.

For spectral library generation, peptides obtained from each tumor section, were analyzed by shotgun proteomics analysis. Here, each MS1 scan was followed by high-collision-dissociation (HCD) of the 20 most abundant precursor ions with dynamic exclusion for 60 s. Total cycle time was ∼2 s. For MS1, 3e^6^ ions were accumulated over a maximum time of 100 ms and scanned at a resolution of 120,000 FWHM (at 200 m/z). MS2 scans were acquired at a target setting of 100,000 ions, accumulation time of 50 ms and a resolution of 15,000 FWHM (at 200 *m/z*). The mass selection window was set to 1.4 Da. Singly charged ions and ions with unassigned charge state were excluded from triggering MS2 events. Besides, the normalized collision energy was set to 28% and one microscan was acquired for each spectrum.

For data-independent acquisition (DIA) analysis, the same LC-MS platform and settings with a few modifications was employed. Specifically, a survey scan at a resolution of 120,000 FWHM (at 200 *m/z*) using a maximum of 5e^6^ ions and 100 ms injection time was followed by 38 DIA mass windows acquired at a resolution of 30,000 FWHM (at 200 *m/z*) accumulating a maximum of 3e^6^ ions and using an automated injection time. The mass range scanned was from 400 to 1,220 *m/z* and stepped normalized collision energy (22.5, 25, and 27.5) was employed. 38 overlapping mass windows (18) splitting each mass window in to equal halves were employed to cover mass range of interest from 400 to 1200 *m/z*.

Because of the fact, we were not able to perform protein quantification before SP3 procedure, for both TMT and DIA runs, peptide injection and mixing (for TMT) were adjusted based on the base peak chromatograms of test injections.

##### Spectral Library Generation for DIA

A spectral library was generated by acquiring 5 shotgun runs (one for each tumor sector). Raw files were processed using MaxQuant (version 1.5.2.8) (19). The search was performed against the human UniProt fasta database (release 2014_07, 20230 entries) using Andromeda search engine (20) with following search criteria: enzyme was set to trypsin with up to 2 missed cleavege; Carbamidomethylation (C) as a fixed modification; oxidation (M) and acetylation (protein N-term) were set as a variable modifications; mass tolerance of 10ppm (precursor ions) and 0.02 Da (fragment ions); minimal peptide length of 7 amino acids. The false discovery rate was set to < 0.01. The spectral library was generated in Spectronaut (Biognosys AG, Schlieren, Switzerland) using default settings.

DIA files were searched in Spectronaut against the generated spectral library using default settings. For quantification only peptides with qvalue < 0.01 and signal to noise ratio (S/N) > 20 were selected and exported.

##### Data Analysis for Microdissected FFPE Samples

Both TMT and DIA data were analyzed using the same R procedures based on the MSnbase package (23). Reporter ion (TMT) and peptide (DIA) intensities were log_2_-transformed and normalized using the vsn package (24). Peptide-level data were summarized into their respective protein groups by taking the median value. For differential protein expression, each patient-sample was treated individually. Protein ratios were calculated for all the protein groups quantified with at least 2 peptides. The R-package “fdrtool” (25) was used to fit a two components model on the median centered log_2_ ratio distributions using the statistic 'normal' (supplemental Figs. S2, S6, and S10). Protein groups with a ratio belonging to the alternative component (q value < 0.2) were considered as differential expressed between the conditions tested.

##### Gene Ontology Enrichment Analysis

Functional enrichment was performed on the list of quantified proteins that were ranked according to the level of differential expression (fold change) using GOrilla (26) followed by GO term redundancy reduction performed by REViGO (27).

##### Protein Solubilization, Digestion and Peptide Desalting for Fresh Frozen Murine HCC

Fresh-frozen tissue samples of murine HCCs (∼60 mg per sample) were homogenized by bead beating in ice-cold PBS using a Precellys24 homogenizer (6000 rpm, 30 s, repeated twice). After a quick spin to remove tissue debris, proteins were solubilized using 4 m urea and 0.1% (v/v) Rapigest (Waters, Milford, MA). Protein digestion was performed using a sequential incubation with LysC (1:50, w/w) (Wako, Neuss, Germany) and trypsin (1:100, w/w) (Promega, Mannheim, Germany), as previously described (28). Digested peptides were desalted using MacroSpin columns (Harvard Apparatus) according to manufacturer instructions.

##### Data Acquisition and Processing for Label-free Quantification (Murine HCC Samples)

For normal liver and tumor samples from mouse model, peptides were measured by data-dependent acquisition on an Orbitrap Velos Pro (Thermo Fisher Scientific) as described before (29). Raw files were processed using MaxQuant (version 1.3.0.5) (19). The search was performed against the mouse Ensembl database (GRCm38.70, 50879 entries) using Andromeda search engine (20) with following search criteria: enzyme was set to trypsin with up to 2 missed cleavage; Carbamidomethylation (C) as a fixed modification; oxidation (M) and acetylation (protein N-term) were set as a variable modifications; mass tolerance of 20 ppm (precursor ions) and 0.5 Da (fragment ions); minimal peptide length of 7 amino acids. The reversed sequences of the target database were used as a decoy database. Peptide and protein hits were filtered at a false discovery rate of 1%. Protein quantification was performed using the label-free quantification (LFQ) function of MaxQuant and the match between run option was selected using a time window of 2 min. LFQ values were extracted from the protein group table, log_2_ transformed and normalized by quantile normalization using the preprocessCore library (30). For each murine HCC, protein fold changes were calculated against an average LFQ value measured from independent normal liver samples obtained from three different mice.

##### Quantification of mtDNA Level by qPCR Analysis

Genomic DNA (including mtDNA) was isolate with QIAamp DNA FFPE tissue kit. (Qiagen). A total of 20 ng was used as a template for qPCR with Sybr Green PCR Mater Mix. qPCR reaction was performed according to the following protocol: 1 × 95 °C - 10 min (DNA denaturation and polymerase activation); 40 × 95 °C −15 s (melting), 60 °C - 1 min (annealing/extension). Mitochondrial DNA abundance was estimated based on mitochondrial genes: *MT-RNR1*; *MT-TL1* and normalized to the gene localized in the nucleus: *B2M*. Each qPCR reaction was performed twice to control for experimental errors. CT values were averaged from two technical replicates. Primers used for the analysis:

MT-RNR1-for: CCACGGGAAACAGCAGTGAT;
MT-RNR1-rev: CTATTGACTTGGGTTAATCGTGTGA;
MT-TL1-for: CACCCAAGAACAGGGTTTGT;
MT-TL1-rev: TGGCCATGGGTATGTTGTTA;
B2M-for: TGCTGTCTCCATGTTTGATGTATCT;
B2M-revTCTCTGCTCCCCACCTCTAAGT

##### Next Generation Sequencing (NGS)

##### Library Preparation and Semiconductor Sequencing

For library preparation, the multiplex PCR-based Ion Torrent AmpliSeq^TM^ technology (Life Technologies) with the Comprehensive Cancer Panel (IonTorrent/Thermo Fisher Scientific, Waltham, MA) covering more than 400 cancer-relevant genes and a modified HCC-specific panel (including 29 genes) were used. Amplicon library preparation was performed with the Ion AmpliSeq Library Kit v2.0 using ∼40 ng of DNA. Briefly, 10 ng DNA were mixed with each of the 4 primer pools, containing all primers for generating ∼16.000 amplicons and the AmpliSeq HiFi Master Mix and transferred to a PCR cycler (BioRad, Munich, Germany). After the end of the PCR reaction, primer end sequences were partially digested using FuPa reagent, followed by the ligation of barcoded sequencing adapters (Ion Xpress Barcode Adapters, Life Technologies). Each individual primer pool was purified using AMPure XP magnetic beads (Beckman Coulter, Krefeld, Germany) and quantified using qPCR (Ion Library Quantitation Kit, Thermo Fisher Scientific) on a StepOne qPCR machine (Thermo Fisher Scientific). The individual library pools were diluted to a final concentration of 100 pm. In total 6 to 8 samples were pooled and processed to library amplification on Ion Spheres using Ion PI™ Hi-Q OT2 200 Kit. Un-enriched libraries were quality-controlled using Ion Sphere quality control measurement on a QuBit instrument. After library enrichment (Ion OneTouch ES), the library was processed for sequencing using the Ion Torrent Hi-Q sequencing 200 chemistry and the barcoded libraries were loaded onto a PI v3 chip and sequenced on an IonTorrent Proton instrument.

##### Variant Calling and Annotation

Data analysis was performed using the Ion Torrent Suite Software (version 4.4.3). After base calling, the reads were aligned against the human genome (hg19) using the TMAP algorithm within the Torrent Suite. Variant calling was performed with the variant caller plugin within the Torrent Suite Software and the IonReporter package using a corresponding bed-file containing the coordinates of the amplified regions. Only variants with an allele frequency > 5% and minimum coverage > 100 reads were taken into account. Variant annotation was performed using Annovar (31). Annotations included information about nucleotide and amino acid changes of RefSeq annotated genes, COSMIC and dbSNP entries as well as detection of possible splice site mutations. For data interpretation and verification, the aligned reads were visualized using the IGV browser (Broad Institute) (32).

## RESULTS

### 

#### 

##### A Protocol That Allows the Quantitative Analysis of ITH in Microdissected FFPE Specimens With Comprehensive Proteomic Coverage

We developed a novel strategy for efficient proteome analysis of microdissected FFPE material. Our goal was to increase protein retrieval and thus the comprehensiveness of proteomic analysis by simplifying existing protocols. To achieve this, we combined a strategy based on heat-induced reversing of formalin fixation with a ultrasensitive and rapid protocol that uses paramagnetic bead technology named SP3 and allows for the removal of multiple mass spectrometry noncompatible reagents, such as for example highly concentrated SDS (9, 33). We confirmed the reproducibility of the LCM combined with SP3 procedure, by comparison of the same tumor region independently microdissected from consecutive slides of a single tumor specimen by shotgun proteomics (typical correlation coefficient R Pearson > 0.95, supplemental Fig. S1).

**Fig. 1.**
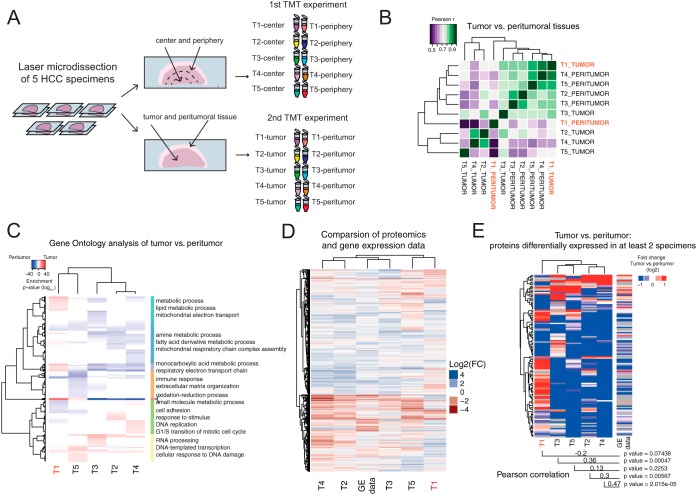
**Spatial tissue proteomics of multiple specimens.** *A*, Schematic representation of proteomics experiments. HCC specimens deriving from 5 patients were microdissected to separate bulk tumor from adjacent peritumoral (nonneoplastic) tissue. Consecutive slices of the same specimens were differentially microdissected to isolate samples deriving either from the center and the periphery of the tumor. Pairs of tumor/peritumoral- and center/periphery-derived tissues were analyzed in two independent TMT-10plex experiments. *B*, Pearson correlation between samples used for neoplastic *versus* nonneoplastic tissue comparison. Unsupervised hierarchical clustering analysis based on proteome profiles separate the two sample groups with the exception of specimen T1 that appears as an outlier. *C*, Ranked gene ontology enrichment of proteins differentially expressed in tumor in comparison to the peritumoral tissue. Blue color indicates proteins enriched in the peritumoral tissue (down-regulated in the tumor), whereas red color corresponds to proteins up-regulated in the HCC. Only terms significant in at least one of the comparisons (*p* value < 0.001) are displayed. Representative terms from each cluster are indicated. *D*, The heatmap shows the comparison of differences in protein abundance (analyzed specimens) and gene expression (from 241 specimens, (15)) between tumor and nontumoral tissue. Only proteins/genes quantified in all data sets were included (3270 cases). Overall proteome changes of the analyzed samples are similar to the changes observed at the gene expression level with the exception of specimen T1 that shows a different protein expression pattern. *E*, Comparison of tumor *versus* peritumor protein expression in HCCs from different patients. The heatmap show protein fold changes for 123 protein groups that were found to be differentially expressed in at least two of the analyzed specimens (q value < 0.2). Transcript level changes observed for the same proteins in an independent HCC cohort are juxtaposed for comparison. The reported Pearson correlation values assess the consistency of each specimen with the reference gene expression profile. For specimens T2:T5 we observed a significant positive correlation between protein and transcript level changes. Related to supplemental Figures S1, S2, and S3, and supplemental Tables S1, S2, and S3.

To spatially quantify the proteome of individual HCCs, we analyzed 5 individual patient samples (for patient characteristics see supplemental Table S1) using a TMT-based quantitative strategy (34). For each specimen, we used LCM to separate bulk tumor from the adjacent peritumoral tissue, and we carefully removed connective tissue forming tumor capsule, fibrous septa, and blood vessels, if present. We designed two quantitative experiments to compare either tumor to peritumoral tissue or the tumor center to its periphery, and used two consecutive slices of the same specimen to perform both analyses (Fig. 1*A*). First, 10 samples (5 tumor and 5 corresponding peritumoral tissue) were combined in a TMT 10-plex experiment. Second, the centers of 5 tumors were separated by LCM from the corresponding peripheries and analyzed together in a separate TMT 10-plex experiment. About half of the peptide material obtained from each spatial region (on average 5–10 μg from approx. 40 mm^2^) was TMT labeled and fractionated using offline high pH reverse phase chromatography (see Methods). The resulting fractions were almost entirely exhausted for a single shotgun mass spectrometry run performed for each fraction.

##### High Spatial Resolution Analysis of Tumor and Nonneoplastic Tissues Identifies Novel Factors Potentially Involved in HCC

We first compared tumor and peritumoral tissues and cross-quantified 5838 protein groups with at least two proteotypic peptides per group (4279 of each were cross-quantified across all 10 TMT channels, supplemental Table S2). Unsupervised hierarchical clustering analysis based on the whole proteome profiles indicated the high similarity of peritumoral tissues and a clear separation from the tumors (Fig. 1*B*), except for one specimen that appeared as an outlier (Tumor 1, see Discussion). We generally observed lower correlation values between tumor samples as compared with peritumoral tissues, indicating a high level of intertumoral heterogeneity. Nevertheless, we were able to detect changes that are consistent across the analyzed samples. Ranked gene ontology enrichment analysis (26) of quantified proteins indicates that functionally related groups of proteins (for example proteins involved in RNA processing and respiratory chain complex component) are differentially expressed in HCC in comparison to the peritumoral tissue. We retrieved Gene Ontology (GO) categories linked to DNA replication and cell cycle to be enriched in the tumor region, possibly reflecting increased cellular growth, however to different extent between specimens (Fig. 1*C*, supplemental Table S3).

To validate the accuracy our findings, we checked whether observed proteomic changes are consistent with gene expression changes that were obtained in a large and independent HCC patient cohort (15) (Fig. 1*D*). Because of the large degree of intertumoral heterogeneity (Fig. 1*B*) each sample was analyzed separately. We defined differentially expressed proteins by analyzing each pair of tumor and nonneoplastic tissue individually and calculated the statistical significance by fitting a two components model on the centered log_2_ ratio distribution (see Methods and supplemental Fig. S2). When we considered proteins that were differentially expressed in at least 2 out of the 5 specimens analyzed (q value < 0.2, 123 proteins in total, supplemental Table S3), we observed a significant positive correlation between our proteome profiles and the gene expression data set derived from 241 HCCs. The correlation with gene expression profiles varied between specimens (R Pearson ranging from 0.13 to 0.47), and as previously observed, Tumor 1 showed an opposite trend with a negative correlation (R Pearson = −0.2). Taken together, these data show that the obtained proteome profiles are informative of the tumor status of the liver tissue and can reveal interpatient heterogeneity that manifests at the protein level (Fig. 1*E*).

Although various protein groups that were affected in abundance were known to be HCC associated, others might have a yet to be defined function in HCC. For instance, we identified Nestin (NEST)—a protein involved in the regulation of G2/M transition—to be up-regulated in the tumor compared with the peritumoral tissue. Consistent with our data, NEST has been reported to be overexpressed in HCC and associated with poor prognosis (35). We also found that multiple components of minichromosome maintenance complex (MCM complex), involved in DNA synthesis and replication during initiation of S phase, are generally up-regulated in analyzed tumors (supplemental Fig. S3*A*). Consistently, the up-regulation of MCM proteins has been previously connected with different cancers including HCC (36). One of the components of the complex, MCM6, was proposed as a novel HCC marker (37). Additionally we observed the general increase of the ribosomal components in the HCC suggesting higher translational activity (supplemental Fig. S3*B*).

At the same time, we consistently observed abundance variation of proteins that (to the best of our knowledge) were not previously linked to HCC, such as *e.g.* Zinc finger protein 207 (ZFP207), which is a kinetochore and microtubule binding protein involved in the mitotic spindle assembly, or fatty acid binding protein 4 (FABP4), which is involved in lipid transport. Therefore, we conclude that our data does not only recapitulate the changes of many known HCC-related factors, but also identifies novel candidate markers for HCC that could be further investigated in the future.

##### Proteomics Analysis Reveals Changes That Could Not Be Detected by Transcriptomic Analysis Alone

**Fig. 2.**
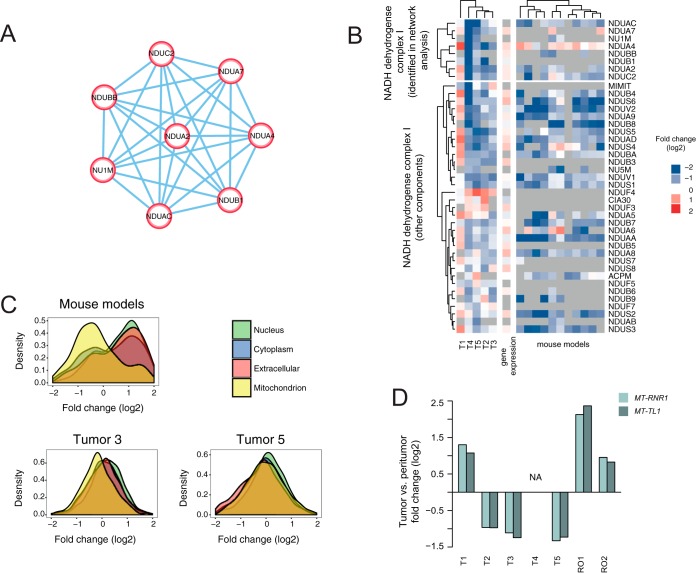
**Specific proteomic changes are not recovered by gene expression analysis.** *A*, The NADH complex I is affected at the protein but not gene expression level in HCC. Network analysis of proteins/genes that were differentially expressed in human HCC at the proteome level, but did not show significant changes at the gene expression level. 148 proteins were selected to derive a STRING protein network (Jensen *et al.* 2009) using confidence score > 0.7. Components of NADH complex I were retrieved as the major cluster from the network analysis. *B*, Heatmap showing the expression changes of proteins identified by network analysis. The down-regulation of NADH dehydrogenase complex I is reflected also in murine models of HCC. The upper panel shows the NADH complex I components that were identified by the network analysis, whereas lower panel displays the remaining components. *C*, Distribution of fold-changes between tumoral and peri-tumoral proteins calculated according to their subcellular localization. In the murine models, we observed a significant shift of mitochondrial proteins toward negative values suggesting a decreased number of mitochondria in tumors as compared with normal livers. In human HCCs, a similar pattern was observed only in 2 out of 5 analyzed specimens with a less pronounced effect size. All the other compartment groups show similar fold change distributions. *D*, Changes in the mtDNA copy number. Barplot showing the fold-change (log2) of mtDNA abundance in tumor compared with peritumoral tissues. To quantify the mtDNA content, two mitochondrial genes (*MT-RNR1* and *MT-TL1*) were analyzed using qPCR (the nuclear gene *B2M* was used for normalization). T1-T5 correspond to the analyzed HCC specimen. The abundance of mtDNA in renal oncocytoma (RO) tumor samples, which are known to have a larger number of mitochondria (41), was also quantified as a positive control. For T4 we were not able to quantify the difference in content of mtDNA because of the lack of peritumoral tissue in the remaining paraffin block of this tumor. T1 in contrasts to other HCC specimens has increased number of mtDNA, which is in line with our proteomics analysis of this particular specimen (see discussion). Related to supplemental Figures S4 and S5.

We next investigated whether the proteomic analysis could reveal additional biological insights that would not emerge from transcriptome data alone. To identify proteins that show robust changes of abundance in HCC, but that are not affected at the level of gene expression, we calculated average fold changes in protein expression (tumor *versus* nonneoplastic tissue) across the analyzed HCCs. Tumor 1 was excluded from this analysis because of its outlier nature (see Fig. 1*B* and Discussion). We used average fold changes to calculate a two components model (as performed for individual HCC analysis), and extracted proteins with q value < 0.2 (considered as differentially expressed, supplemental Fig. S2*F*). We then retained only hits that, while being differential expressed at the protein level, showed no or modest difference in gene expression (148 hits in total, supplemental Fig. S4). Network analysis revealed that these proteins are functionally related. Among the down-regulated proteins, we found an enrichment for the mitochondrial NADH dehydrogenase complex I, possibly indicating a difference in subcellular compartmentalization between tumor and nonneoplastic tissues that is not reflected in gene expression data (Fig. 2*A*).

To test this hypothesis under well-defined conditions and on a larger cohort of samples, we acquired proteomic data from genetically defined HCC mouse models closely resembling human hepatocarcinogenesis (39, 16). We quantitatively compared 11 fresh frozen murine HCC samples to normal murine livers using label free quantification (see Methods). Consistently with human HCC specimens, we also detected a strong decrease mitochondrial of NADH dehydrogenase complex I across all the murine models tested (Fig. 2*B*). We next classified the measured proteins according to their subcellular localization and compared the distribution of the calculated fold changes. In case of murine models, we noticed a significant decrease in the amount of mitochondrial proteins in the tumor part that could explain the decreased abundance of NADH dehydrogenase complex I. However, in case of human HCCs, we observed a much less pronounced, nevertheless still significant, decrease of mitochondrial proteins in only half of the analyzed specimens (Fig. 2*C*, supplemental Fig. S5). This was not consistent with the decrease of NADH dehydrogenase complex I that was detected in most of the specimens (on average 2-fold; Fig. 2*B*). To check whether our proteomics data reflects differences in the subcellular compartmentalization in HCC, we assessed the abundance of mitochondria in the analyzed specimens using an independent approach. The most informative methods of estimating mitochondrial content are based on assays measuring directly mitochondrial activity in cells or tissue extracts (39). Unfortunately, FFPE tissues are not suitable for such analyses because of fixation. We therefore decided to quantify the mitochondrial DNA (mtDNA) content as a proxy for mitochondria abundance, bearing in mind those other factors may also influence this quantity. Alterations in mtDNA copy number are in fact a common feature of multiple cancer types, however they do not always correlate with the changes in the expression of mitochondrial proteins (40).

We compared the ratio mtDNA/nuclear DNA (nDNA) between tumoral and peritumoral tissues using qPCR. For the analysis we selected two genes encoded by mtDNA (*MT-TL1* encoding 12S rRNA and *MT-RNR1* encoding tRNA-leu) and normalized them to the nuclear gene *B2M*. To validate the assay accuracy, we analyzed samples of renal oncocytoma - a tumor type that has been previously reported to contain a higher number of mitochondria (41). For all the samples analyzed except for tumor 1, we observed around 2-fold decrease of the mtDNA/nDNA ratio (for both reporter genes), which is in line with our proteomics data (Fig. 2*D*). These data confirm a reduced mitochondrial content in tumor regions as compared with the surrounding hepatic parenchyma.

Taken together, our data show that proteomic analysis of tumor samples can (1) reveal changes in protein abundance that do not manifest at the gene expression level, (2) detect broad alterations in subcellular compartmentalization between normal and cancer cells, (3) identify a decreased level of NADH dehydrogenase complex I in HCCs that likely represent a metabolic rearrangement of the tumor cells consistent with the Warburg effect (42, 43).

##### Clinically Relevant Proteins Show Intratumoral Heterogeneity

**Fig. 3.**
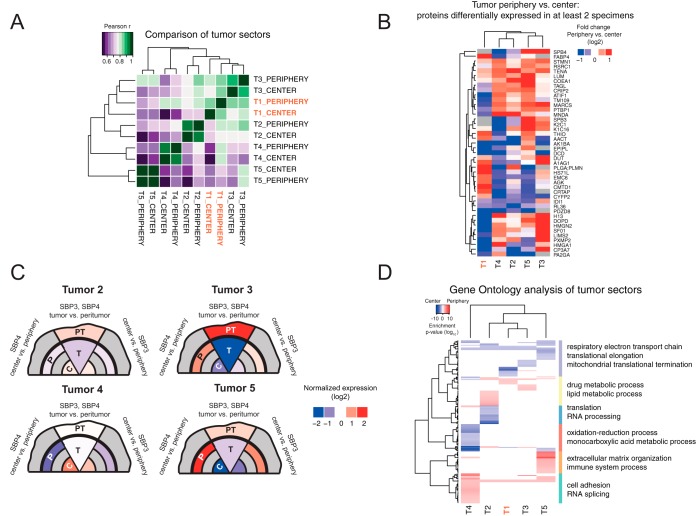
**Proteome-level intratumoral heterogeneity in HCCs is highly patient specific.** *A*, Pearson correlation between samples used for ITH analysis (comparison of tumor center *versus* periphery). Separate clustering of different tumors indicates a prominent interpatient variability, however significant differences between tumor sectors can be detected (*B*). The heatmap shows the expression change between tumor center and its periphery. Only proteins significantly affected in at least 2 HCC specimens are shown (q value < 0.2). *C*, Graphical representation of normalized Serpin B4 and Serpin B3 expression across tumor regions. Each tumor has been divided into three sections corresponding to the expression of Serpin B3 (center and periphery) on the left side, Serpin B4 (center and periphery) on the right side and Serpin B3 and Serpin B4 together (tumor *versus* peritumor, both proteins are merged because they were identified in the single protein group in corresponding experiment). The observed spatial pattern is highly variable. *D*, Ranked gene ontology enrichment of proteins differentially expressed across tumor sectors. Blue color indicates proteins enriched in the center of the specimen, red color corresponds to proteins enriched at the tumor periphery. Only terms significant in at least one of the comparisons (*p* value < 0.001) are displayed. Related to supplemental Figures S6 and S7, and supplemental Tables S2 and S4.

Next, we investigated the level of ITH across multiple patients and analyzed proteomes of two distinct tumor regions from the same five specimens used for tumor *versus* nonneoplastic tissue comparison (Fig. 1*A*). Five thousand six hundred fifty-nine protein groups were quantified with at least two proteotypic peptides per group (4275 of which were cross-quantified across all the 10 TMT channels, supplemental Table S2). As expected, unsupervised hierarchical clustering based on the correlation of the proteome profiles revealed that differences across tumors are much more pronounced than the variations within the same specimen (Fig. 3*A*). To identify proteins that are differentially expressed across tumor regions in multiple specimens, we analyzed each pair of tumor sectors individually as performed for tumor *versus* nonneoplastic tissues (see Methods and supplemental Fig. S6). We then extracted 43 protein groups that were significantly changed in at least two of the analyzed specimens (q value < 0.2, Fig. 3*B*, supplemental Table S4). Among these, two of the most striking examples of proteins previously shown to be clinically relevant for HCC are SerpinB3 (SPB3) and SerpinB4 (SPB4), also known as squamous cell carcinoma antigens (SCCAs). We found both proteins to be homogenously expressed across tumor regions only in one of the five tumors analyzed. In other specimens we observed strong changes in protein abundance that were always consistent between SPB3 and SPB4, with both proteins being in some cases more abundant at the tumor periphery and in others at the tumor center (up to 16-fold, Fig. 3*C*). These data suggest that the ITH of SCCAs might be independent from the tumor geometry, but rather derive from other, yet to be characterized cues. It is worth mentioning that these proteins were previously proposed as histological markers of HCC (44). Our analysis shows that the diagnostic outcome may drastically differ if different sectors of tumor are analyzed.

To gain functional insight into protein expression across tumor regions, we performed ranked GO enrichment analysis (26) for each specimen. Except for a group of biological processes linked to mitochondrial metabolism that we found to be down-regulated at the tumor periphery in multiple specimens, we generally observed distinct biological functions to be affected in different tumors (Fig. 3*D*). These functions are often related to protein synthesis, cell adhesion, and energy and drug metabolism. For instance, we found the microtubule-interacting protein stathmin (STMN1) and myristoylated alanine-rich protein kinase C substrate (MARCS) to be increased at the tumor periphery in most of the analyzed specimens (supplemental Fig. S7*A* and S7*B*). Both these proteins are required for tumor cell migration in HCC (45, 46), and STMN1 has been proposed as a negative prognostic marker in liver cancer (47). Thus, these findings indicate that spatial proteomic ITH analysis provides an additional level at which functionally/prognostically relevant proteins in HCC should be verified. Such analysis can be used to pinpoint potentially more robust diagnostic markers that are equally expressed in all tumor sectors and show strong difference in bulk tumor *versus* peritumoral tissue comparison (supplemental Fig. S7*C* and S7*D*).

Bearing in mind the limitations derived from the small number of cases that we analyzed, we conclude that spatial proteomic ITH is detectable across multiple HCC specimens. However, differently from tumor *versus* nonneoplastic comparison, we observed more often patient-specific effects than general changes present in all HCCs. This manifested in the same proteins showing different patterns of ITH in different patient samples (Fig. 3*C*). Our data suggests that the presence of proteome-level ITH might contribute an additional layer of interpatient variability. The example of SCCAs expression clearly indicates that proteomic ITH affects also proposed diagnostic and prognostic markers, and thus deserves further investigation in HCC as well as other cancer types.

##### Individual Tumor Specimen Analysis at High Spatial Resolution Visualizes Expression Gradients That Are Not Reflected On the Genetic Level

**Fig. 4.**
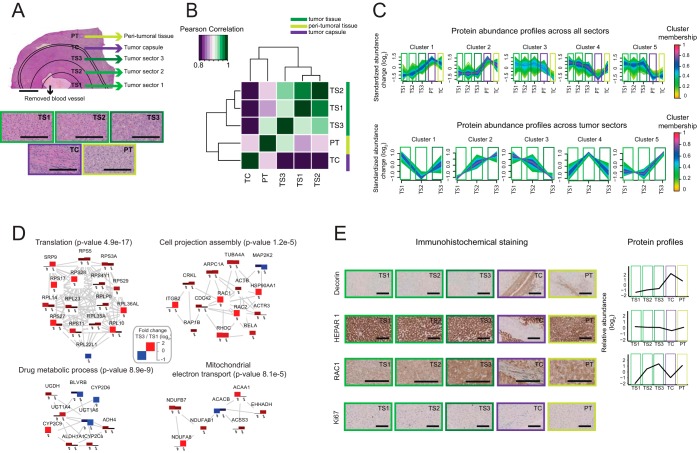
**Highly resolved spatial proteomics of HCC specimen.** *A*, Macroscopic picture of a micro-dissected HCC (scale bar is 5 mm). The blood vessel localized in the center of the tissue was removed. Five tissue sectors were collected for proteomic analysis: three morphologically identical sectors of the tumor (TS1, TS2, TS3) - based on the distance to the center of the specimen; the tumor capsule (TC); and the peri-tumoral tissue (PT). Below microscopic pictures of micro-dissected, H&E-stained (40x) regions indicating morphological homogeneity across tumor sectors (TS1, TS2, TS3) are presented. Differences to the TC and PT regions are apparent; nuclei appear dark. Scale bars are 200 μm. *B*, Pearson correlation between different sectors based on the TMT data set. The different sectors have distinct proteomic signatures. *C*, Soft clustering analysis of HCC spatial proteome by the Fuzzy c-means algorithm (48). Color code represents membership values consistency of expression profiles within a given cluster. The upper panel includes all measured sectors (including capsule and peritumoral tissue). Profiles from the bottom panel were calculated only from the tumor sectors. The optimal number of clusters was estimated using the “elbow” method by plotting the minimum centroid distance against the number of clusters (49). Cluster 2 contains the highest number of proteins with high membership values and indicates a subset of proteins that gradually increase their abundance from the center of the specimen (TS1) toward its periphery (TS3). *D*, Network analysis of differentially expressed proteins. In total, 230 protein groups were selected to derive a protein network using STRING (61) using confidence score > 0.7. Network modules were then extracted in Cytoscape using MCODE (62) and module functional enrichment was assessed using ClueGO (63) using the whole list of quantified proteins as background. In the network representation, the log2 transformed TS3/TS1 ratio obtained from the DIA and TMT methods are juxtaposed and shown in red for increased and blue for decreased proteins. Brighter colors indicate stronger effects. For display purposes, the range of log2 ratio value was limited between −1 and 2. *E*, Consecutive slides of analyzed specimen were stained with antibodies against that showed different expression pattern and compared with their expression profiles measured by qMS (log2 scale). Decorin (20x) - highly enriched in connective tissue (mainly tumor capsule), HEPAR 1 antigen (20x) - equal expression across all sectors, RAC1 (40x) - increase of expression from the center toward the periphery of the specimen), Ki67 (20x). Scale bars are 200 μm. Related to supplemental Figures S8 and S9, and supplemental Tables S5 and S6.

We next decided to push the boundaries of spatial analysis on an individual HCC specimen as it might typically occur during the clinical routine. We selected a well-differentiated tumor specimen and used FFPE slides of 10 μm thickness and 2 cm in diameter from half of the encapsulated spherical solid tumor (including fibrous tissue) for LCM (Fig. 4*A*). The accuracy of microdissection allowed removing necrotic areas and prominent blood vessels located in the center of the tumor to avoid contamination with unrelated cell types. Because no overt heterogenic subpopulations of tumor cells could be detected by conventional microscopy, we divided the tumor into three concentric sectors (TS1, TS2, and TS3) based on the distance to the center of the specimen. Additionally, we separated the tumor capsule (TC) and peritumoral tissue (PT) and compared their proteomes with the tumor itself. Using TMT labeling, we quantified 2863 protein groups with at least two proteotypic peptides across these sectors (supplemental Table S5). To test the compatibility with an alternative proteomic workflow, we also used label-free analysis based on data-independent acquisition (DIA) (18) to analyze an independently micro-dissected sample from the same tumor block. We obtained similar results (supplemental Fig. S8), and almost the same coverage (2722 protein groups cross-quantified across tumor sectors, supplemental Table S5). Notably, this analysis was performed without peptide fractionation step, and required less material per spatial sector. Because only about one tenth of the obtained peptide material has been used for MS analysis, it is conceivable to improve proteomic coverage or even to further increase the spatial resolution in the future using this approach.

High Pearson correlation (>0.9) between the proteome profiles obtained from the different tumor and peritumor sectors indicated that most of the proteins were expressed at comparable levels across the whole specimen (Fig. 4*B* and supplemental Fig. S9*A*). Although this finding is consistent with the fact that we chose to study a well-differentiated HCC with its morphology highly reminiscent of the adjacent nontumorous parenchymal liver tissue, we identified clusters of proteins showing distinct abundance profiles across the specimen and clearly discriminating neoplastic and nonneoplastic hepatocellular tissue (Fig. 4*B* and supplemental Fig. S9*B*). The proteome of the connective tissue forming the tumor capsule appeared very different because of the presence of high abundant extracellular matrix proteins such as collagens, Fibrillin and Decorin.

Soft clustering analysis of HCC spatial proteome by the Fuzzy c-means algorithm (48) performed within the tumor (TS1-TS3) indicates that a prominent subset of proteins show changes in their abundance at the tumor periphery as compared with the inner part (Figs. 4*C* and supplemental Fig. S9*B*). The optimal number of clusters was estimated using the “elbow” method by plotting the minimum centroid distance against the number of clusters (49). We performed functional network analysis of proteins that were up- or down-regulated at the tumor periphery and showed consistent fold changes in both independent analyses (DIA and TMT). We found a strong enrichment in ribosomal components among proteins up-regulated at the peripheral sectors of the specimen. Interestingly, we also found a network of proteins that are involved in the regulation of cell migration (which is in line with initially analyzed specimen, Fig. 3*D*) showing a similar abundance profile (Fig. 4*D*). These include the small GTPases RAC1 and CDC42, controlling the formation of lamellipodia and filopodia, regulators of actin cytoskeleton dynamics, such as Actin-related protein 3 (ACTR3), and proteins that are involved in the regulation of cell migration at the extracellular level, such as Integrins beta 1 (ITGB1) beta 2 (ITGB2). We validated some of our findings by IHC staining performed on the same specimen (Fig. 4*E*). For this analysis we included Decorin that shows increased expression in the tumor capsule, Hepar 1 antigen (50) that is evenly expressed across the specimen and RAC1 that shows the gradient of expression from the tumor center to the periphery of the specimen. To rule out different proliferation rates between the tumor center and the periphery potentially affecting the proteomic profiles we performed an immunohistochemical Ki67-staining. As shown in Fig. 4*E*, we observed a similar low proliferation rate (<2%) across the sectors.

To test if the variations of protein abundance coincide with genomic variability as detectable by a commonly used gene panel sequencing approach, we analyzed the tumor sectors and peri-tumorous tissue by targeted Next Generation Sequencing (NGS). As described before for 25% of HCCs (13), no mutation was detectable in well-defined liver cancer-related genes (*e.g.* TP53, ARID1A, CTNNB1, and AXIN1, among others). We only found mutations in the following three genes: *DNA (cytosine-5)-methyltransferase 3A (DNMT3Ap.Tyr247Phe*),*Myosin Heavy Chain 11 (MYH11p.Leu1563Pro*), and *Cyclin-dependent kinase 12 (CDK12p.His369Arg*). Because these mutations showed largely similar allele frequencies (∼30%) in all of the tumor sectors (except for CKD12) (supplemental Table S6), we do not expect a substantial impact onto the spatial tumor proteome. We conclude that the analysis of different tumor regions revealed that intratumoral heterogeneity is apparent at the proteome level, even in case of a specimen that is largely homogenous both at the morphological and genetic level, as determined by targeted NGS.

## DISCUSSION

The aim of the presented study was to comprehensively analyze the spatial proteome of HCC on multiple levels. First, we compared neoplastic and nonneoplastic tissue microdissected from 5 FFPE specimens of HCC of different etiology. We provide a comprehensive resource of the HCC proteome by integrating gene and protein expression changes of human HCCs with alterations in protein expression observed in multiple murine HCC models of different, cancer-relevant, genetic background. We identified 755 proteins that show consistent expression changes (with the same sign) except for one tumor entity. The observed differences for this specimen are higher than expected from just an interpatient heterogeneity. Careful post-analysis examination of the specimen and clinical records revealed that the corresponding patient received transarterial chemoembolization (TACE) treatment before the surgery. We therefore speculate that the unusual proteome profile of this specimen may be a consequence of the preoperative treatment. However, further investigations are required to characterize the impact of chemoterapeutic treatment on the HCC proteome *in vivo*.

The set of identified proteins includes multiple factors that have been already connected to HCC, but also some that have not been studied in context of liver tumorigenesis. We observed strong enrichment of ribosomal components, suggesting increased translational activity within neoplastic hepatocellular tissue. In fact, multiple oncogenic pathways have been shown to induce (de)-regulation of translational machinery, and increased protein synthesis is required to promote cellular transformation (51). These data suggest that increased translational activity facilitates HCC development.

We also identified functionally related proteins, such as NADH dehydrogenase complex I members, to be down-regulated in HCC at the protein level without being affected at the level of gene expression. Additionally, in some of the analyzed specimens, we observed a general decrease of mitochondrial proteins indicating that the total number of mitochondria in HCC is lower than in normal hepatocytes. This is supported by a corresponding decrease in mtDNA content in tumor regions as compared with adjacent tissues. A potential explanation for the reduced abundance of mitochondria is an increased mitophagy activity that selectively removes defective mitochondria from the tumor cells. In the future, it would be worthwhile to investigate further the impact of mitophagy inhibition on HCC development, as suggested for other cancer types (52). However, it is important to consider that the observed reduction in mitochondrial proteins and mtDNA content could also derive from a rearrangement of the mitochondrial network and structure in cancer cells, as observed in aging tissues (53).

Second, we identified proteins responsible for intratumor proteome heterogeneity in HCC and highlight the relevance of ITH for biomarker discovery studies and diagnostics. ITH is a well-characterized feature of many tumors, which to date has been mainly investigated at the morphological and genetic level. Here we identified proteins that are differentially expressed between the center and the periphery of the same tumor, indicating spatially defined metabolic and functional heterogeneity of tumor cells. We identified several proteins that are significantly changed in multiple specimens of HCC, however the spatial patterns of these changes are not always consistent across the examined tumors. For example, ribosomal proteins are not only affected by ITH, but they are also one of the most prominent features up-regulated in HCCs. The same is true for the fatty acid binding protein-4 (FABP4), which is known to be involved in other diseases, such as obesity, diabetes, atherosclerosis and cardiac dysfunction (54), but to the best of our knowledge has not been studied in context of HCC. According to our proteomics data, FABP4 appears to be overexpressed in the tumor in comparison to the nonneoplastic tissue, and at the same time displayed an uneven distribution across sectors in two of the analyzed specimens (up to 9-fold in one of the tumors).

Our comparative analysis indicates that proteomic ITH in HCC is, at least in part, functionally related to the alterations that distinguish tumor and nonneoplastic tissues. This observation might indicate the existence of heterogeneous cancer cell populations that are confined in spatially defined regions of the tumor. These different cell populations might at least to some extent resemble the nonneoplastic tissue of origin, and therefore display a variable sensitivity to drug treatment.

In conclusion, we describe a strategy that can be universally applied to FFPE samples to investigate the abundance of thousands of proteins in solid tumors with excellent spatial resolution. Our strategy can be used jointly with other approaches based *e.g.* on MALDI imaging (55–59) or epitope-based tissue imaging (60), to deconvolute the complexity of tumor specimens and help incorporate ITH information in the design of novel diagnostic and therapeutic strategies.

## DATA AVAILABILITY

The mass spectrometry proteomics data have been deposited to the ProteomeXchange Consortium (http://proteomecentral.proteomexchange.org) (21) via the PRIDE partner repository (22) with the data set identifier PXD007052.

## Supplementary Material

Supplemental Data

## Footnotes

^1^ The abbreviations used are:

IHC: immunohistochemistry
FFPE: formalin fixed paraffin embedded
ITH: intratumoral heterogeneity
LCM: laser capture microdissection
qMS: quantitative MS
MCM: minichromosome maintenance
RO: renal oncocytoma.
