# Supplementary material for: Spatial Tissue Proteomics Quantifies Inter- and Intratumor Heterogeneity in Hepatocellular Carcinoma (HCC)
Source: Mol Cell Proteomics. 2018 Jan 23;17(4):810–25. doi: 10.1074/mcp.RA117.000189 (PMC5880102; doi:10.1074/mcp.RA117.000189)
Supplement: Supplemental Data [file supp_17_4_810__index.html]

Spatial Tissue Proteomics Quantifies Inter- and Intratumor Heterogeneity in Hepatocellular Carcinoma (HCC) — Supplemental Data 

# Spatial Tissue Proteomics Quantifies Inter- and Intratumor Heterogeneity in Hepatocellular Carcinoma (HCC)

## Supplemental Data

- Supplemental Materials - Supplemental Materials to: Spatial tissue proteomics quantifies inter- and intra-tumor heterogeneity in hepatocellular carcinoma
- Supplemental Table 2 - Complete expression table for all experiments (6 analyzed tumors, murine models and gene expression)
- Supplemental Table 1 - Samples characteristics
- Supplemental Table 3 - Tumor vs. peritumor comparison
- Supplemental Table 4 - Intratumoral heterogeneity table.
- Supplemental Table 5 - Complete TMT and DIA proteomic datasets for the specimen analyzed with high spatial resolution.
- Supplemental Table 6 - Cancer gene panel sequencing dataset
- Supplemental Table 7 - Detailed information about identified proteins and peptides.
