# Supplementary material for: Spatial Tissue Proteomics Quantifies Inter- and Intratumor Heterogeneity in Hepatocellular Carcinoma (HCC)
Source: Mol Cell Proteomics. 2018 Jan 23;17(4):810–25. doi: 10.1074/mcp.RA117.000189 (PMC5880102; doi:10.1074/mcp.RA117.000189)
Supplement: Supplemental Data [file supp_RA117.000189_4790_2_supp_58830_p2t9fy.pdf]

Supplemental Materials to:

Spatial tissue proteomics quantifies inter- and intra-tumor heterogeneity in hepatocellular carcinoma

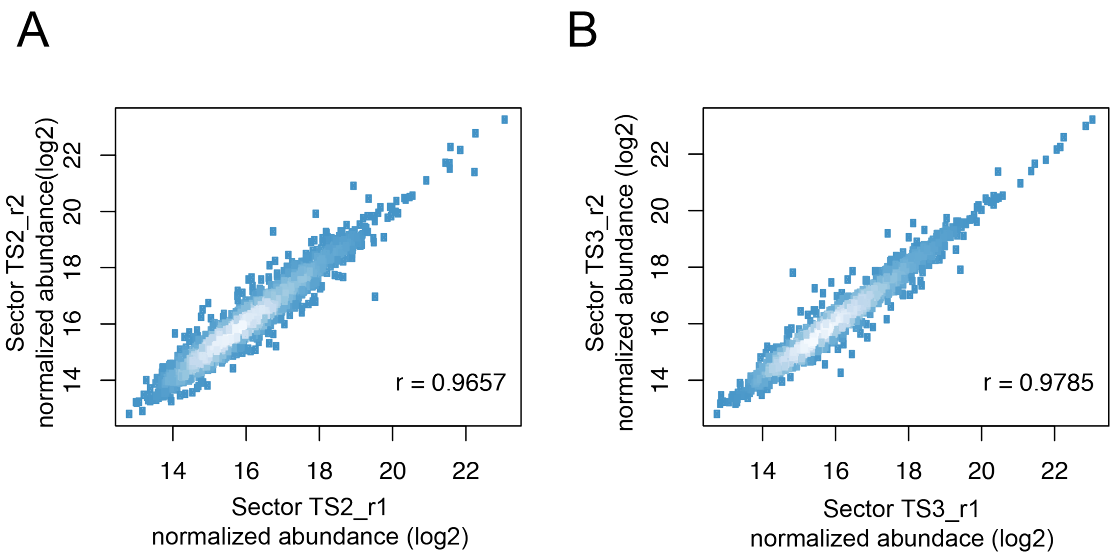

Figure S1. Reproducible protein quantification of independently microdissected regions of the same FFPE specimen.

Two consecutive slides of a HCC specimen were micro-dissected (specimen presented of Figure 4) and subjected to the described sample preparation procedure followed by mass spectrometry analysis. Quantitative proteome profiles of the same sectors (A- Sector TS2, B- sector TS3) from consecutive slides of the same tissue block are highly correlated, indicating high reproducibility of the sample preparation procedure. Normalized abundance was derived from log2 transformed intensities normalized using the vsn package (52).

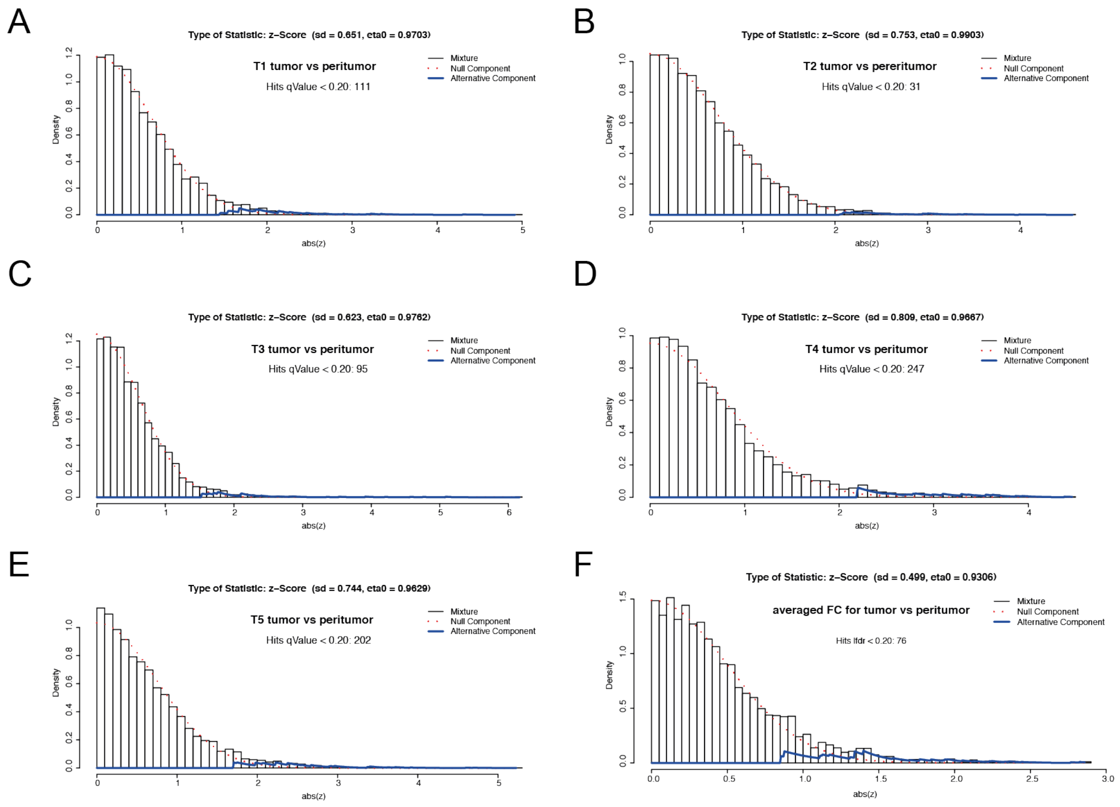

Figure S2. Statistical models for tumor vs, peritumor comparisons.

Histograms and the densities of the fitted two-component models (null component = not differential expressed; alternative component = differentially expressed proteins) are shown. The models were fitted on median centered $\log_2$ ratios (z). The estimated parameters of the models (sd = standard deviation of the normal distribution; eta0 = estimated proportion of null p-values) are reported in the plot title. The models and graphical outputs were generated with the R package 'fdrtool' ((54), see Methods). (A) T1 tumor vs peritumor, (B) T2 tumor vs peritumor, (C) T3 tumor vs peritumor, (D) T4 tumor vs peritumor, (E) T5 tumor vs peritumor. (F) averaged fold changes (averages were calculated without T1).

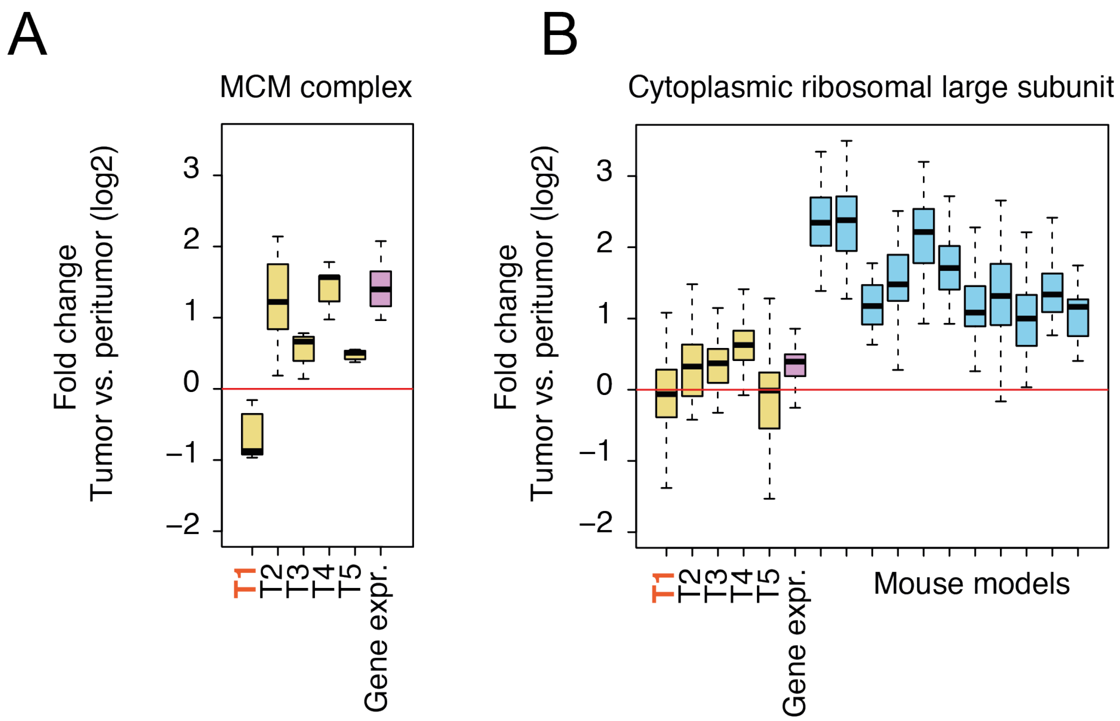

Figure S3. Complexes with differential expression.

Boxplots showing the fold changes of complexes with differential expression across multiple specimen in comparison to non-tumorous tissue (yellow: FFPE specimens; pink: gene expression data (15); blue: murine models (23)). (A) Distribution of MCM complex components. With the exception of the tumor 1 we observed a general increase of expression of MCM complex members. (B) Distribution of cytoplasmic ribosomal large subunit. We identified slight increase of expression of ribosome components in the analyzed human tumor specimens. This effect was much more pronounced in murine HCCs.

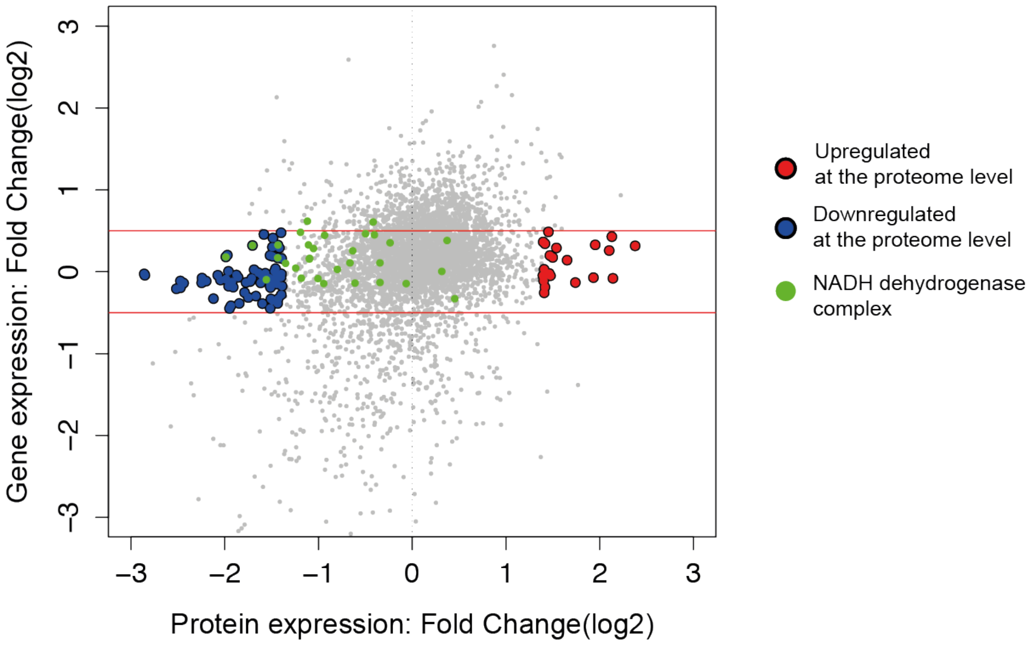

Figure S4. Changes detected at the proteome level but not at the gene expression level.

We compared changes of gene expression (15) and protein abundance (average of analyzed tumors) between HCCs and adjacent non-neoplastic liver tissue. The highlighted points indicate proteins that are not affected at the transcript level but are either upregulated (in red) or downregulated (in blue) at the proteome level. Green points correspond to NADH dehydrogenase complex I components.

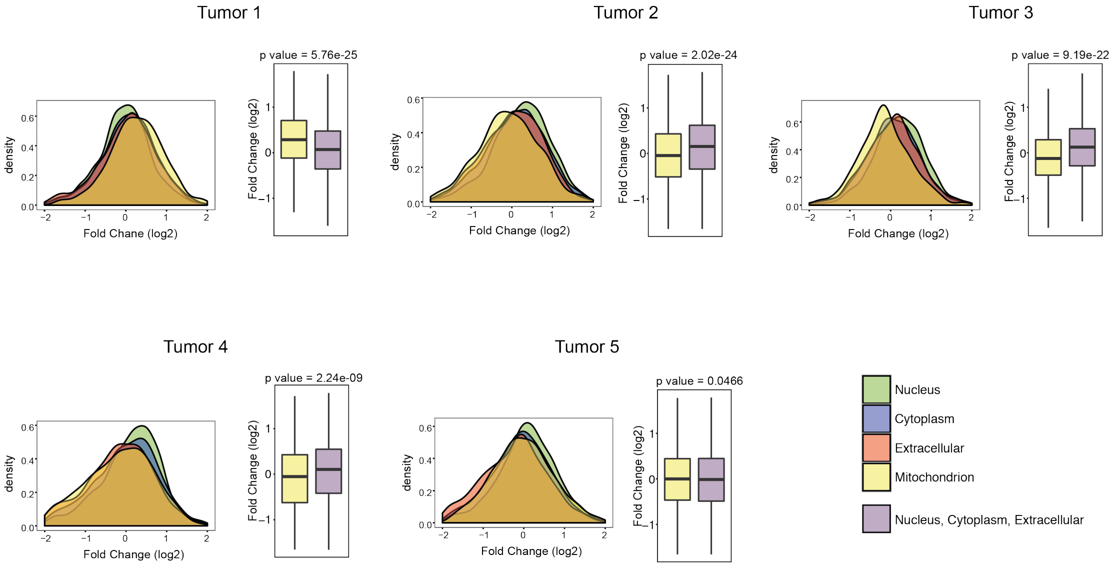

Figure S5. Distribution of fold-changes for different cellular compartments.

Density plots shows the distribution of fold-changes between tumoral and peri-tumoral proteins calculated according to their subcellular localization for all analyzed tumors. Barplots show the difference in fold change of mitochondrial proteins and proteins localized in all other compartments.

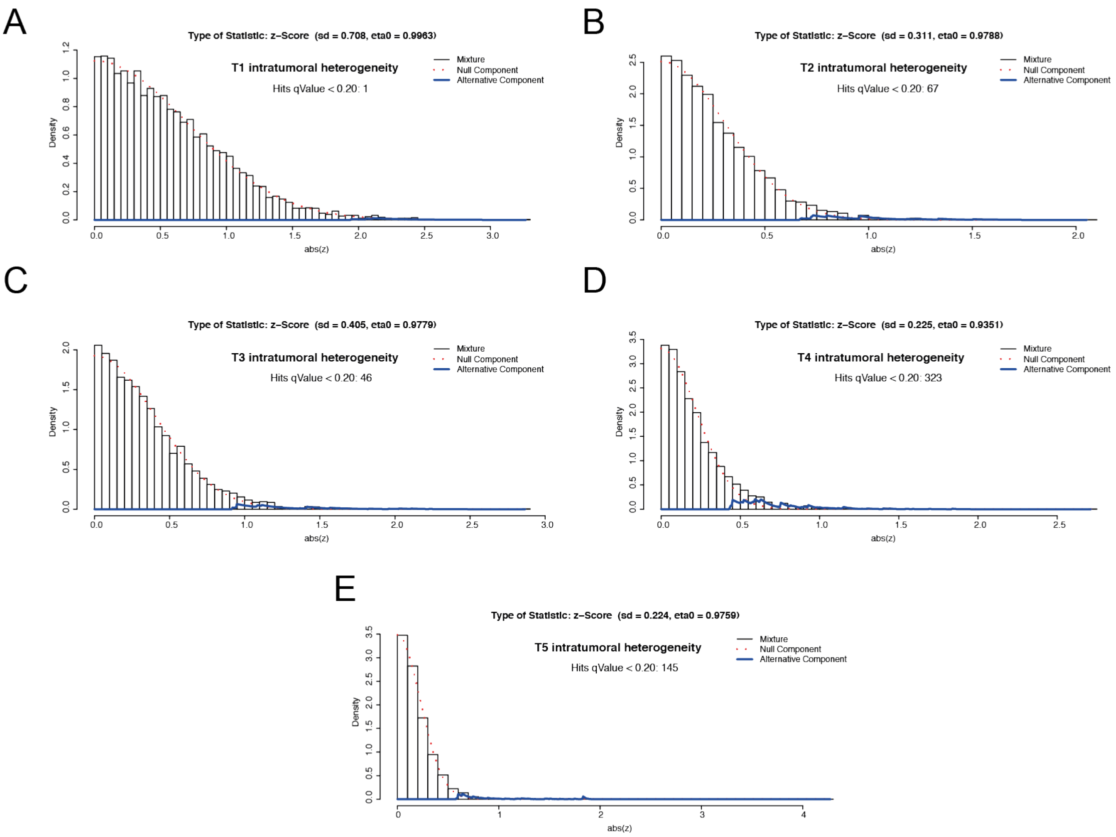

Figure S6. Statistical models for periphery vs, center comparisons.

Histograms and the densities of the fitted two-component models (null component = not differential expressed; alternative component = differentially expressed proteins) are shown. The models were fitted on median centered $\log_2$ ratios (z). The estimated parameters of the models (sd = standard deviation of the normal distribution; eta0 = estimated proportion of null p-values) are reported in the plot title. The models and graphical outputs were generated with the R package 'fdrtool' ((54), see Methods). (A) T1 intratumoral heterogeneity, (B) T2 intratumoral heterogeneity, (C) T3 intratumoral heterogeneity, (D) T4 intratumoral heterogeneity, (E) T5 intratumoral heterogeneity.

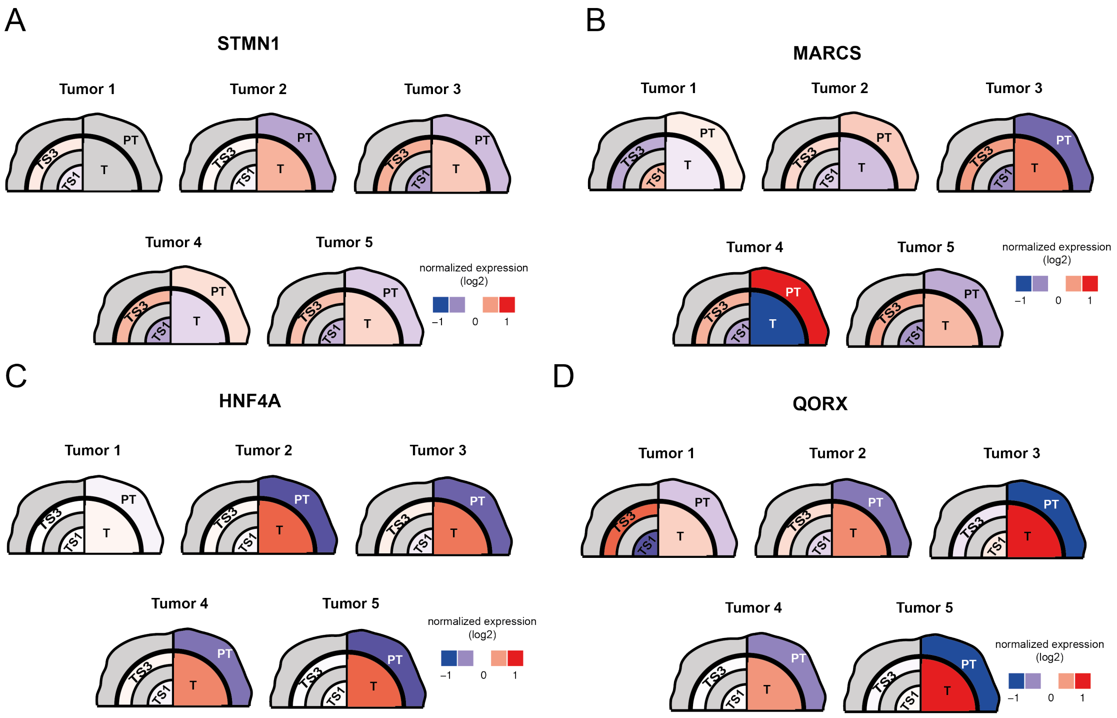

Figure S7. ITH affects clinically relevant proteins.

Graphical representation of (A) Stathmin (STMN1), (B) Myristoylated alanine-rich C-kinase substrate (MARCS), (C) Hepatocyte nuclear factor 4-alpha (HNF4A), (D) Quinone oxidoreductase PIG3 (QORX) normalized expression across the analyzed specimen Each tumor has been divided into two sections, corresponding to the expression within the tumor (TS1 vs. TS3) on the left side and expression in bulk tumor and adjacent peritumoral tissue (T vs. PT) on the right side.

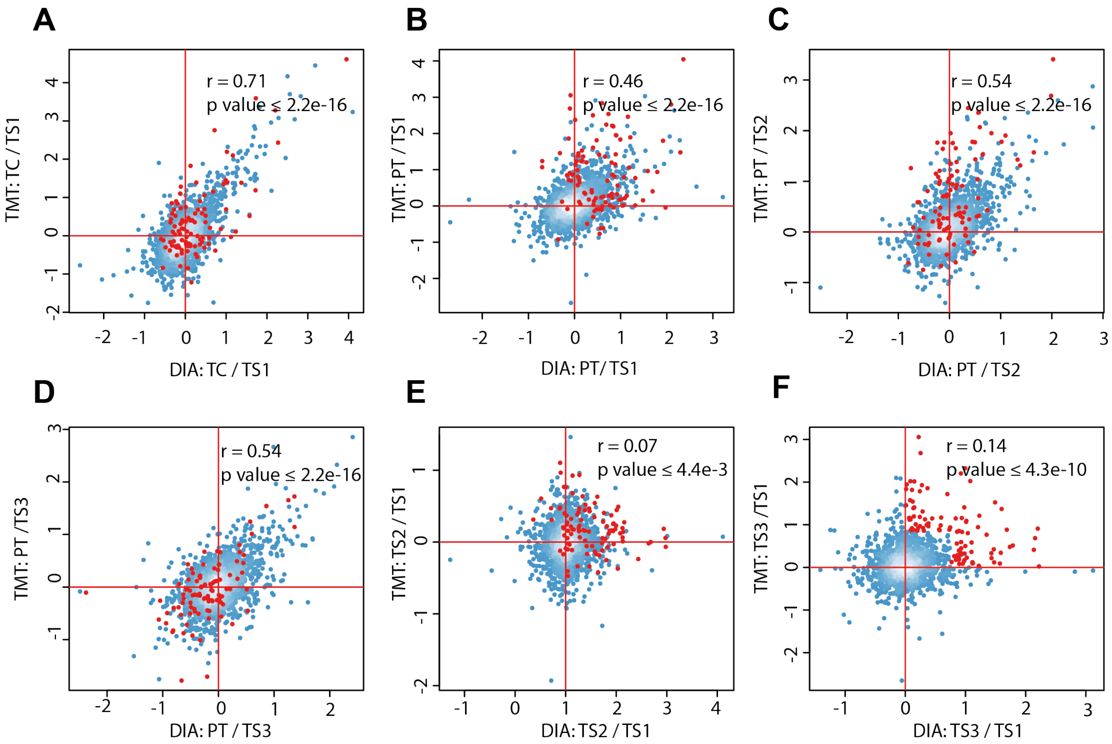

Figure S8. Comparison of protein fold-changes measured using DIA and TMT methods.

In order to verify the consistency between the DIA and TMT datasets, log_2-transformed fold-changes calculated for the different sectors were compared and representative examples are shown (A-F). Significant positive correlation was observed between the fold changes obtained using the two different quantitative strategies. Differences between non-tumor, either tumor capsule (TC) or peri-tumor (PT), and tumor sectors (TS) are more pronounced and display a good degree of linearity in the estimated fold changes (A-D). Differences between tumor sectors (TS1-TS3) are less pronounced and, therefore, the fold changes estimated by TMT and DIA display positive, but lower correlation values (E and F). Notably, the correlation between TS3/TS1 fold changes (F) is increased as compared to TS2/TS1 (E). This suggests that the most peripheral tumor sector (TS3) has a more distinct proteome profile as compared to the other tumor sectors. This is consistent with the soft clustering analysis that indicates clusters of protein being differentially expressed at the tumor periphery (Figure 1D). In order to visualize this, we highlighted proteins as red dots that were identified as expressed at higher levels at the tumor periphery. These proteins show a trend of increasing abundance that becomes more pronounced at the tumor periphery (TS3) as compared to the inner tumor sectors (compare E and F). Only proteins with at least two peptides identified were included in the analysis.

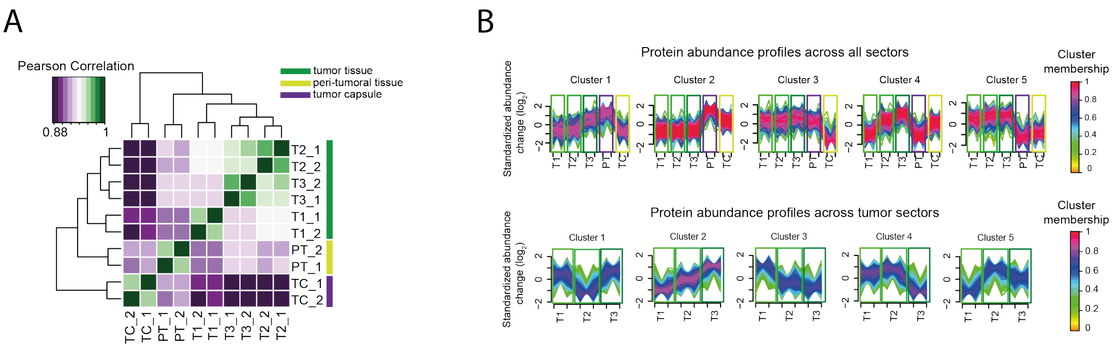

Figure S9. Spatial proteomics analysis based on the single sample DIA dataset.

(A) Pearson correlation between different sectors based on the DIA dataset. Each sample was measured in two technical replicates. The different sectors have distinct proteomic signatures. (B) Soft clustering analysis of HCC spatial proteome by the Fuzzy c-means algorithm (34). The optimal number of clusters was estimated using the “elbow” method by plotting the minimum centroid distance against the number of clusters (35). Color code represents membership values consistency of expression profiles within a given cluster. The upper panel includes all measured sectors (including capsule and peritumoral tissue). Profiles from the bottom panel were calculated only from the tumor sectors. Cluster 2 contains the highest number of proteins with high membership values and indicates a subset of proteins that gradually increase their abundance from the center of the specimen (TS1) towards its periphery (TS3).
